# Multi-Parametric Cerebellar Neuroimaging in Subjective Cognitive Decline as Predictors of Cognitive Alterations in Alzheimer’s Spectrum

**DOI:** 10.1192/bjo.2025.10242

**Published:** 2025-06-20

**Authors:** Shailendra Mohan Tripathi, Richa Shukla, Porimita Chutia

**Affiliations:** 1 King George’s Medical University, Lucknow, India; 2 Institute of Medical Sciences, University of Aberdeen, Aberdeen, United Kingdom

## Abstract

**Aims:** Subjective Cognitive Decline (SCD) represents the earliest, reversible stage in Alzheimer’s dementia (AD) spectrum, marked by self-appraised cognitive deterioration, that escapes objective detection. The intricate fine-tuning of cognition by cerebellum has been substantiated by the Cerebellar Cognitive Affective Syndrome and “Dysmetria of thought” theory. However, the role of cerebellum in SCD is understated in research. This study aims to determine the relationship between cerebellar neuroimaging parameters and cognition in patients with SCD.

**Methods:** Patients with SCD, with a Clinical Dementia Rating score of 0, were assessed on Addenbrooke’s Cognitive Examination-III (ACE). Multiparametric MRI (Volumetric analysis of cerebellum, Diffusion tensor imaging at Middle Cerebellar Peduncles (MCP), Magnetic resonance spectroscopy (MRS)) was carried out. Relationship between cognition and neuroimaging parameters was determined.

**Results:** A total of 28 SCD patients with a mean age of 70.89±3.89 years were included. There were significant positive correlations of attention with axial diffusivities (AxD) at bilateral MCP; fluency with right cerebellar white matter volumes (CWMV); visuospatial function with left CWMV and fractional anisotropy (FA); total ACE scores with total CWMV, bilateral AxD. Significant negative correlations of myoinositol/creatine (mI/Cr) with attention, fluency and memory were revealed on MRS. Linear regression analysis exhibited significant associations between total ACE scores and CWMV; attention and axial diffusivities; memory and right cerebellar volume; fluency and CWMV, Cerebellar cortical volumes; Visuo-spatial function and FA at left MCP.

**Conclusion:** The aforementioned significant relationships highlight the unique role of multiparametric neuroimaging in early detection of cerebellar ultrastructural alterations, and the modulatory impact of cerebellum in cognition during initial stages of AD continuum. Furthermore, longitudinal studies are warranted to predict long term cognitive outcomes in SCD using cerebellar neuroimaging parameters.

